# Case Report: Urachal calculus with an atypical intravesical CT appearance and literature review

**DOI:** 10.3389/fruro.2026.1899074

**Published:** 2026-08-25

**Authors:** Xingqiang Long, Yunli Zhi, Yinghe Zhou, Daoqi Wang, Haixiang Guo

**Affiliations:** Department of Urology, The Second Affiliated Hospital of Kunming Medical University, Kunming, China

**Keywords:** case report, clinical features, computed tomography, urachal calculi, urachus

## Abstract

**Background:**

Urachal calculi are uncommon complications of urachal remnants. Their clinical and radiological appearances overlap with bladder calculi, vesicourachal diverticula, infected urachal cysts, and calcified urachal tumors, creating a potential diagnostic pitfall.

**Case description:**

A 54-year-old man presented with intermittent lower abdominal pain lasting more than six months without hematuria, dysuria, umbilical discharge, fever, or other lower urinary tract symptoms. Cystoscopy at a local hospital showed bladder inflammation but no stone. In our hospital, computed tomography demonstrated a 1.2 x 0.9 cm densely calcified lesion (approximately 1112 HU) at the junction of the bladder dome and anterior wall. The lesion appeared to lie within the bladder outline and initially mimicked a bladder diverticular calculus. Repeat cystoscopy at our institution identified a partially intravesical calculus with a component embedded in the urachal tract. The intravesical component was fragmented using a holmium laser, followed by laparoscopic partial cystectomy and en bloc excision of the urachal remnant and residual calculus. Histopathological examination showed an inflamed urachal remnant without intestinal metaplasia, dysplasia, or malignancy. The urinary catheter was removed after 2 days, and the patient was discharged after a 7-day hospitalization. Post-discharge recurrence could not be assessed because the patient was lost to follow-up.

**Conclusion:**

A midline calcification at the bladder dome should prompt consideration of a urachal origin even when it appears intravesical. Multiplanar CT assessment, systematic inspection of the bladder dome, and histological evaluation of the excised remnant are important for diagnosis and management.

## 1 Introduction

The urachus is a fibromuscular tubular remnant connecting the bladder dome to the umbilicus, derived from the cloaca and allantois (1). Failure of complete urachal obliteration during infancy results in urachal anomalies, which include patent urachus (approximately 50% of all urachal malformations), urachal sinus (approximately 15%), vesicourachal diverticulum (3%–5%), and urachal cyst (approximately 30%) (2, 3). These anomalies may be complicated by infection, malignant transformation, or stone formation (4). We report a urachal calculus that presented with isolated lower abdominal pain, was missed during an initial cystoscopic examination, and appeared intravesical on CT. We compare this presentation with previously reported cases and discuss the mechanisms, differential diagnosis, diagnostic approach, surgical management, prognosis, and recurrence risk. Selected published cases are compared with the present case in **Table 1**.

**Table 1:** Patient–level comparison of selected published urachal calculus or urachal remnant calcification cases and the present case.

Case	Age/sex	Presentation	Imaging findings	Stone location	Treatment	Pathology	Follow – up
Nargund, 1994 (5)	59/M	Lower abdominal pain	Radiopaque suprapubic shadows, anterior bladder relation	Urachal sac near anterior bladder	Excision and stone removal	Inflamed urachus	Asymptomatic at 4 years
Ansari, 2002 (6)	55/M	Irritative voiding symptoms	Fixed anterior–wall focus, CT showed a midline tract and pedunculated intravesical extension	Urachovesical tract	Combined endoscopic and laparoscopic removal with urachal excision	NR	NR
Milotic, 2002 (7)	Adult/F, age NR	Dysuria, infraumbilical mass	8 x 4 x 3 cm cyst with multiple shadowing calculi	Inflamed urachal cyst	Surgical excision	Inflamed urachal cyst	NR
Atalar, 2016 (8)	24/M	Hematuria and pelvic pain	Ultrasound and multidetector CT showed a tubular tract with a stone	Vesicourachal diverticulum	Diverticulum and stone removed, bladder defect repaired	Histological confirmation	Uneventful, good clinical condition
Rawal, 2022 (9)	44/M	Incidental finding during trauma imaging	CT, sagittal reformation delineated the median umbilical ligament	Urachal remnant calcification appearing within bladder	No intervention, discharged	NR	NR
Naiem, 2022 (10)	26/M	Periumbilical pain, discharge, erythema, fever, and localized peritonitis	Ultrasound and contrast–enhanced CT	Infected urachal cyst/sinus with urachaolith	Open excision and umbilicoplasty	Infected urachal cyst, no malignancy	Uneventful, regular activity at 6 weeks
Mirzaei, 2025 (1)	54/M	Current renal colic from ureteral stones, remote umbilical urinary discharge	CT incidentally showed a 6–mm urachal stone	Proximal patent urachus near umbilicus	No intervention for the urachal stone	NR	Urological follow–up recommended
Present case	54/M	Isolated intermittent lower abdominal pain	1.2 x 0.9 cm, 1112 HU, apparent intravesical location, no diverticular neck, local inflammatory change	Urachovesical junction with embedded urachal component	Holmium laser lithotripsy plus laparoscopic partial cystectomy and urachal excision	Inflamed urachal remnant, no intestinal metaplasia, dysplasia, or malignancy	Lost to follow–up, recurrence not assessable

NR, not reported.

## 2 Case report

### 2.1 Patient information

A 54–year–old man was admitted with intermittent dull lower abdominal pain that had persisted for more than six months. The pain did not radiate and was not related to urination. He denied urinary frequency, urgency, dysuria, gross hematuria, lumbar pain, fever, chills, and umbilical discharge.

### 2.2 Clinical findings and diagnostic assessment

The patient had previously undergone cystoscopy at a local hospital, where bladder inflammation was reported but no calculus was identified.

On admission, the umbilicus was normal without discharge, erythema, or a visible sinus. Mild suprapubic tenderness was present without a palpable mass.

Urinalysis showed red blood cells but no pyuria, and urine culture was negative. Procalcitonin was 0.061 ng/mL, interleukin–6 and C–reactive protein were reported as not elevated. Renal and liver function tests were within the institutional reference ranges.

**Figure 1:**
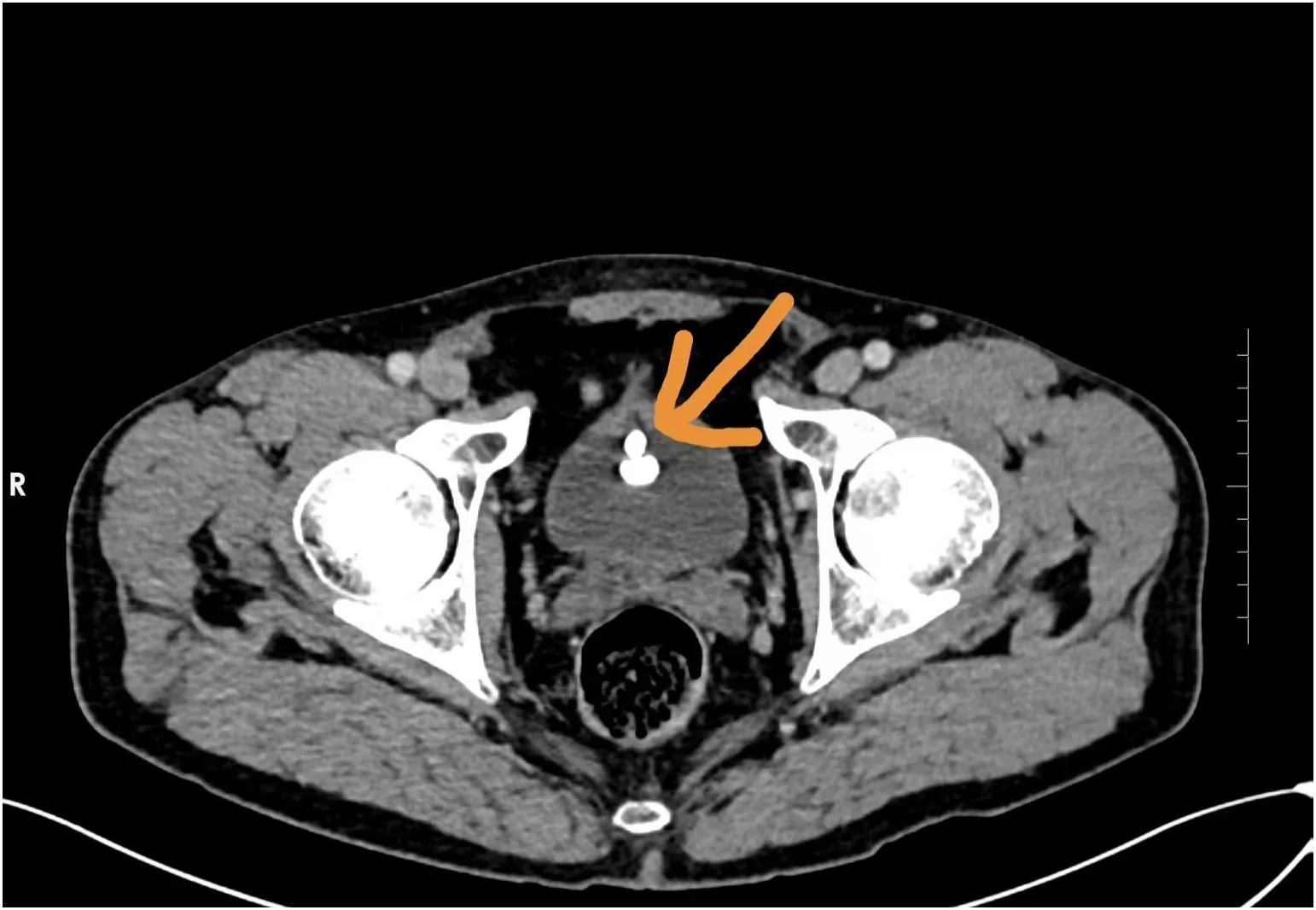
Axial CT image showing a densely calcified nodular lesion at the junction of the bladder dome and anterior wall (arrow), measuring approximately 1.2 x 0.9 cm with an attenuation of approximately 1112 HU.

Non–contrast CT demonstrated a 1.2 x 0.9 cm densely calcified nodular lesion at the anterior bladder wall near the dome, with an attenuation of approximately 1112 HU (**Figure 1**). The lesion appeared to lie within the bladder outline, and no separate diverticular neck was identified. Urachal stones are rare, have diverse clinical manifestations, and are easily misdiagnosed or missed.

### 2.3 Therapeutic intervention

**Figure 2:**
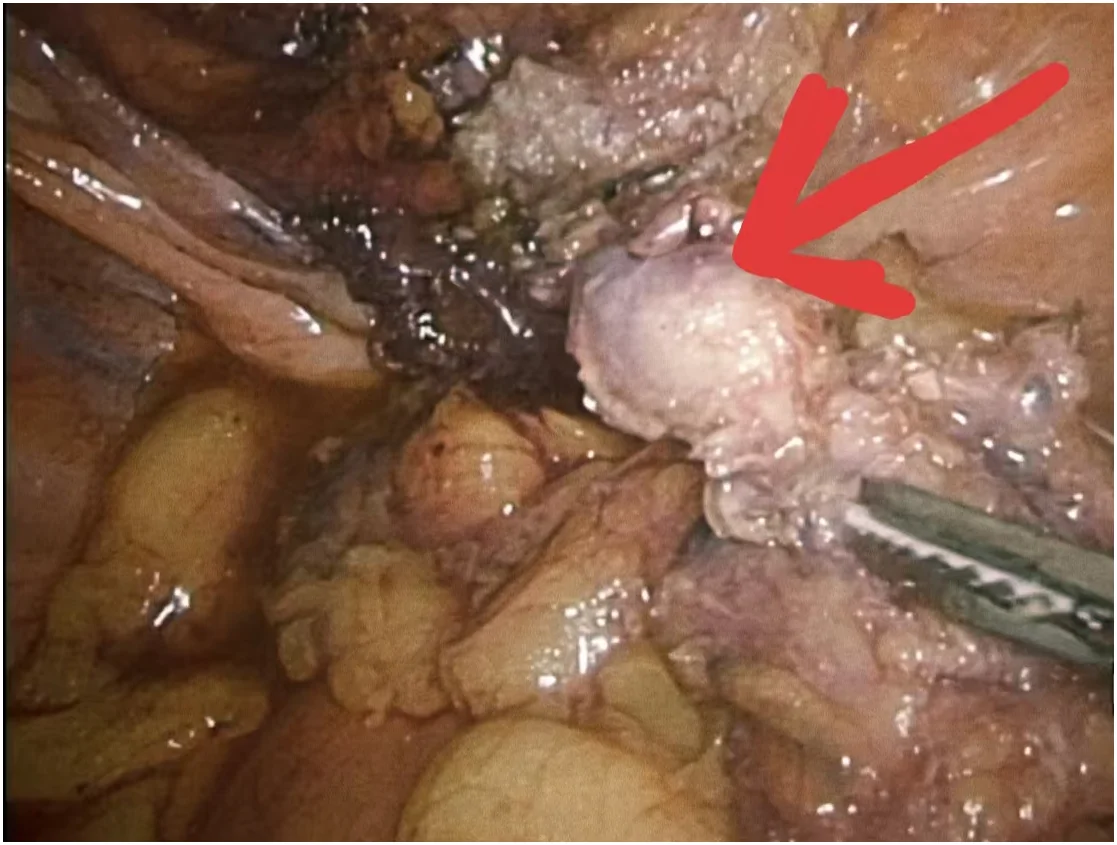
Intraoperative view showing the urachal remnant at the bladder dome and the impacted urachal calculus.

After completing preoperative evaluation and obtaining informed consent, the patient underwent combined transurethral and laparoscopic surgery under general anesthesia. Repeat cystoscopy systematically evaluated the bladder neck, trigone, ureteric orifices, lateral walls, anterior wall, and dome. A calculus was identified at the junction of the bladder dome and anterior wall, with an intravesical component and a residual component embedded in the urachal tract (**Video 1**). Holmium laser lithotripsy was used to fragment and remove the visible intravesical component. Due to the inaccessible location of the residual stone embedded within the urachus and concerns regarding potential recurrence and malignant transformation, the procedure was converted to laparoscopic resection. A cord–like urachal remnant was identified at the bladder dome, and the embedded calculus was palpable within the tract (**Figure 2**). The involved urachal remnant, residual calculus, and a cuff of the bladder dome were excised en bloc using an ultrasonic device, followed by bladder closure.

### 2.4 Pathology, follow–up, and outcomes

**Figure 3:**
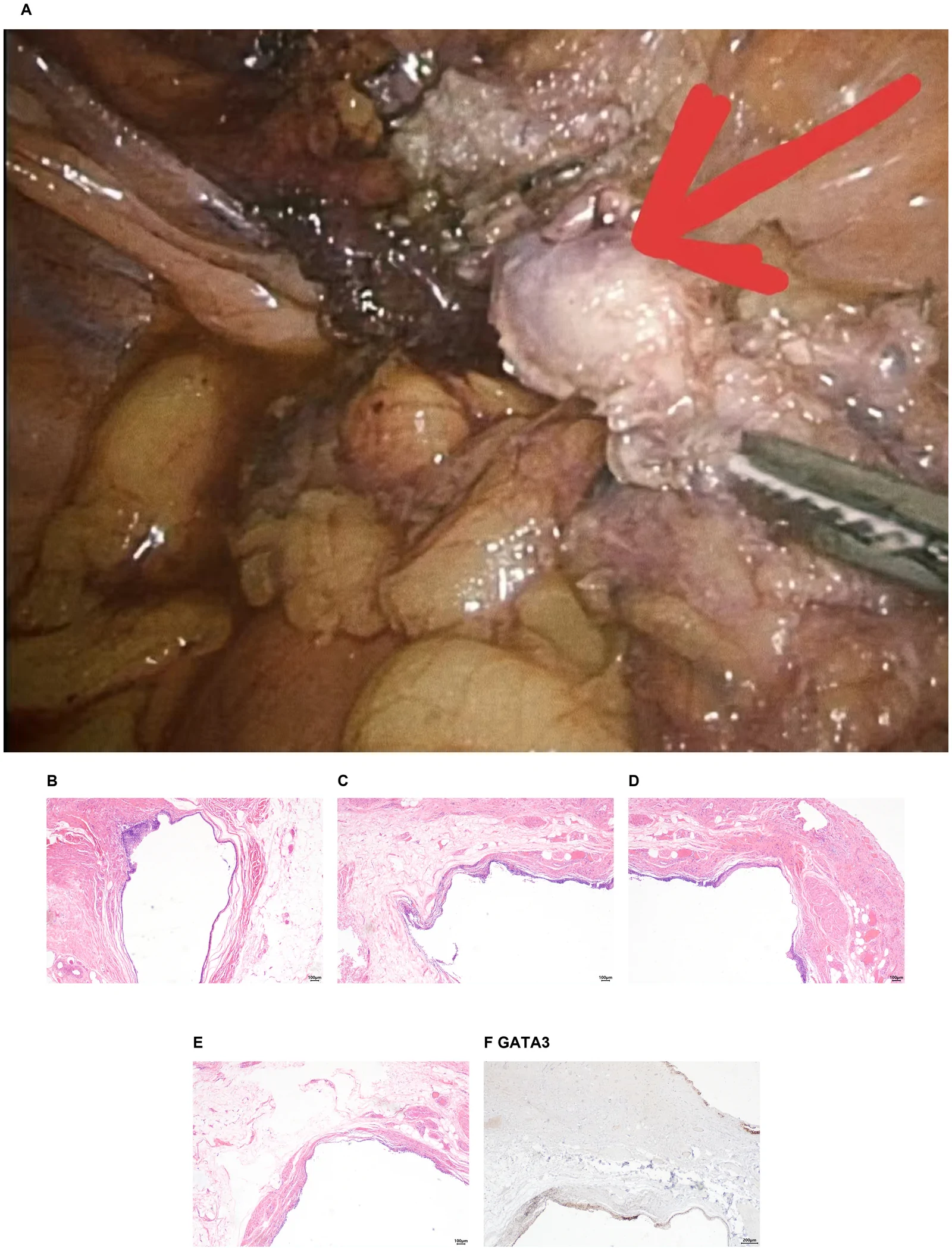
**(A)** Intraoperative view showing the urachal remnant at the bladder dome and the impacted calculus. **(B–E)** Hematoxylin and eosin–stained sections show inflammation associated with calculus formation. **(F)** GATA3 immunohistochemical staining. No intestinal metaplasia, dysplasia, or malignancy was identified.

Histopathological examination confirmed a urachal remnant associated with calculus formation and inflammation. No intestinal metaplasia, dysplasia, or malignancy was identified (**Figure 3**). The urinary catheter was removed after 2 days, and the total hospital stay was 7 days. The patient recovered sufficiently for discharge. The patient was subsequently lost to follow–up, no post–discharge imaging or cystoscopy was available, and symptom status and recurrence could not be assessed. The clinical timeline is summarized in **Table 2**.

**Table 2:** Clinical timeline of the present case.

Relative time	Clinical event
>6 months before admission	Intermittent lower abdominal pain developed without urinary or umbilical symptoms.
Before referral	Local cystoscopy showed bladder inflammation but no calculus, CT showed a 1.2 x 0.9 cm calcified lesion at the bladder dome/anterior wall.
At our institution	Repeat cystoscopy identified a partially intravesical calculus with a component embedded in the urachal tract.
During admission	Holmium laser lithotripsy was followed by laparoscopic partial cystectomy and en bloc excision of the urachal remnant and residual calculus.
Postoperative day 2	The urinary catheter was removed.
Postoperative day 7	The patient was discharged, postoperative complications were not documented in the records available for this revision.
After discharge	The patient was lost to follow–up, symptoms and recurrence could not be assessed.

## 3 Structured literature review

### 3.1 Search approach

A structured search of PubMed was updated through 18 July 2026 using combinations of ‘urachal stone’, ‘urachal calculus’, ‘urachal calculi’, ‘urachovesical calculus’, ‘vesicourachal diverticulum’, and ‘urachal calcification’. Reference lists of relevant reports were also screened.

Individual human reports were retained when a urachal calculus or a calcified urachal remnant was described and patient–level diagnostic or treatment information was extractable. Reports of urachal malignancy without a true calculus, animal cases, duplicates, and records without extractable clinical information were excluded. Because of heterogeneous terminology and incomplete reporting, this was treated as a structured literature review rather than a systematic review.

### 3.2 Comparison with reported cases

**Table 1** summarizes the published cases with the most directly extractable information and includes the present case for comparison. NR indicates information not reported in the PubMed record or the source available to the authors.

## 4 Discussion

### 4.1 Clinical novelty and comparison with previous cases

The value of the present case lies in the combination of an atypical symptom profile and a misleading anatomical appearance, rather than in the surgical technique alone. A previous report described a 55–year–old man with irritative voiding symptoms whose CT showed a midline urachal tract containing a cylindrical calculus that extended into the bladder as a pedunculated stone, that patient was also treated using combined endoscopic and laparoscopic techniques (6). In contrast, our patient had isolated intermittent lower abdominal pain, no lower urinary tract or umbilical symptoms, and an initially negative cystoscopic examination. The calcification appeared to lie within the bladder outline, without an obvious tubular tract or diverticular neck on the available CT images. These features created a realistic risk of misclassification as a bladder diverticular calculus.

### 4.2 Epidemiology and pathogenesis

Urachal abnormalities are rare congenital anomalies with an incidence of approximately 2 per 100,000 hospitalized adults (11)While urachal cysts and urachal carcinoma represent the most common urachal pathologies in adults, urachal calculi are exceptionally rare (2, 3). The formation of urachal calculi is thought to result from prolonged retention of residual urine within the urachal tract, combined with repeated urine reflux, urinary tract inflammation, epithelial cell shedding, and urate deposition (1, 12) The relatively narrow lumen of urachal remnants and the alkaline urinary environment may further promote stone formation and growth (4).

### 4.3 Differential diagnosis

The differential diagnosis of a densely calcified lesion at the anterior bladder wall includes several important entities that must be carefully considered (**Table 3**). In our case, the key distinguishing features were: (1) the midline location at the bladder dome, (2) the very high CT attenuation (1112 HU), consistent with a calculus rather than tumor calcification, (3) the absence of soft tissue enhancement on contrast CT, and (4) intraoperative confirmation of the stone being embedded at the base of the urachus.

**Table 3:** Differential diagnosis of anterior bladder wall calcification.

Condition	Similarity to urachal calculi	Key distinguishing features
Bladder diverticular stones	Location at bladder wall, calcified density	Diverticulum communicates with bladder lumen, stones typically mobile, changes in size after voiding on CT
Distal ureteral stone	Peri–bladder calcified lesion	Proximal ureteral dilatation, hydronephrosis, stone in ureteral course rather than midline
Urachal carcinoma with calcification	Midline supravesical mass	Soft tissue component with enhancement, irregular margins, calcification within tumor matrix
urachal cyst or infection	Midline supravesical lesion	cystic or inflammatory component, wall thickening, surrounding fat stranding

### 4.4 Diagnostic approach

Ultrasonography can identify a fixed echogenic focus with posterior acoustic shadowing and may show a cystic remnant. CT provides a broader assessment of the lesion’s relationship to the bladder dome, median umbilical ligament, surrounding soft tissue, and any enhancing component. Sagittal and coronal reformations are particularly useful when axial images make the lesion appear intravesical (13–15) Cystoscopy remains important for assessing the bladder mucosa and intravesical component, but a small lesion at the anterior dome can be overlooked. A systematic inspection of the dome and correlation with cross–sectional imaging are therefore essential.

### 4.5 Surgical management

Management should be individualized according to symptoms, infection, communication with the bladder, the possibility of malignancy, and the extent of the remnant. Symptomatic or complicated adult urachal remnants are commonly managed by excision, often with a bladder cuff when the lesion involves the dome (6, 13). Transurethral lithotripsy may remove an exposed intravesical component but can leave an embedded calculus and the underlying urachal remnant. In the present case, the combined approach allowed endoscopic clearance of the intravesical component and laparoscopic en bloc removal of the residual calculus and involved remnant. The resection also provided tissue for definitive histological exclusion of malignancy.

### 4.6 Prognosis, recurrence, and limitations

Published case reports generally describe favorable outcomes after removal of the calculus and treatment of the underlying urachal abnormality. Nargund and Donaldson reported no symptoms at four years after excision (5), whereas most other reports provide only short or incompletely described follow–up. The small number of heterogeneous cases does not permit a reliable recurrence rate. Complete removal of an involved remnant is biologically plausible as a means of reducing recurrent stasis and calculus formation, but long–term surveillance should be guided by pathology and the completeness of resection. In the present case, the patient was lost to follow–up after discharge. No follow–up imaging or cystoscopy was available, and symptom status and recurrence therefore could not be assessed. However, the long–term outcomes of stone recurrence and malignant transformation in our patient who did not follow medical advice for follow–up after surgery are still unclear.

## 5 Conclusion

Urachal stones are not only rare, but also have diverse clinical manifestations and are easily misdiagnosed and missed diagnosis. This case highlights that urachal stones may exhibit atypical CT features—specifically, location within the bladder outline rather than in the classic extravesical position—which can lead to misdiagnosis as bladder diverticular stones. When clinicians encounter midline calcified lesions at the anterior bladder wall, even with atypical imaging features, the possibility of urachal calculi should be considered. A high index of clinical suspicion, combined with careful CT evaluation and thorough cystoscopic examination are essential for accurate diagnosis. Complete surgical resection of the urachal remnant with partial cystectomy remains the recommended treatment to prevent recurrence and potential malignant transformation.

## Data Availability

The original contributions presented in the study are included in the article/**Supplementary Material**, further inquiries can be directed to the corresponding author/s.

## Supplementary material

Supplementary Video 1 Cystoscopic examination showing a calculus at the junction of the bladder dome and anterior wall, with a component embedded in the urachal tract.
